# 

**Published:** 1909-01

**Authors:** 


Chronicles of the Octopus: Evolution of the Great Medical Trust. Medical Politics in Epigram (Lydston). Proserpine Again.

VOL. XXIV. JANUARY, 1909. No. 7.

LIBRARY
SURGEON GENERAL'S OFFICE
JAN. 19 1909

### TEXAS MEDICAL JOURNAL

"RED BACK"

PUBLISHED AT AUSTIN, TEXAS, BY
F. E. DANIEL, M. D., - - - Proprietor
PRINTED BY THE VON BOECKMANN-JONES CO.

Subscription, $1.00 a Year, in Advance. - - - Single Copies, 10 Cents

Entered at the Postoffice at Austin, Texas, as Second-class Mail Matter

